# Comment on Tabet et al. Vestibular Migraine versus Méniere’s Disease: Diagnostic Utility of Electrocochleography. *Audiol. Res.* 2023, *13*, 12–22

**DOI:** 10.3390/audiolres14010003

**Published:** 2023-12-25

**Authors:** Jeremy Hornibrook

**Affiliations:** Department of Otolaryngology and Audiology, Christchurch Hospital, Christchurch 8011, New Zealand; jeremy@jhornibrook.com

I wish to comment on an aspect of the recently published article by Saliba I. et al., titled Vestibular Migraine versus Meniere’s Disease: Diagnostic Utility of Electrocochleograpy [1]. It is commendable that the authors would use the EcochG to make a distinction, even though the technique is becoming unfashionable with the increased use of MRI inner-ear imaging. To be fair, the authors do concede in their discussion that there may be an electrocochleography (EcochG) technique more appropriate than the one they used. The truth of this cannot be overstated.

The EcochG technique employed was with a remote (eardrum) electrode and a click stimulus. It has been demonstrated that responses from a remove electrode are significantly smaller than those from a direct electrode. Also, the diagnostic sensitivity from clicks is vastly inferior to tone bursts, whose responses cannot be reliably measured by a remote electrode. The fact that audiologists have not employed the tone burst stimulus EcochG seems to be because it requires specialty co-operation and customized equipment that cannot be achieved by the standard commercial systems used by audiologists. This information has been exhaustively reviewed [2,3,4]. The variation in EcochG techniques and the results have likely impeded progress in the objective diagnosis of Meniere’s disease and its distinction from vestibular migraine for at least twenty years.
